# Attitude of nurses towards palliative care and its associated factors in Ethiopia, systematic review and meta-analysis

**DOI:** 10.1186/s12904-024-01402-w

**Published:** 2024-03-08

**Authors:** Addisu Getie, Manay Ayalneh, Tigabu Munye Aytenew, Melaku Bimerew, Adam Wondmieneh

**Affiliations:** 1 Department of Nursing, College of Medicine and Health Sciences, Debre Markos University, Debre Markos, Ethiopia; 2 Department of Nursing, College of Medicine and Health Sciences, Injibara University, Injibara, Ethiopia; 3 Department of Nursing, College of Medicine and Health Sciences, Debre Tabour University, Debre Tabour, Ethiopia

**Keywords:** Attitude, Palliative care, Nurses, Ethiopia

## Abstract

**Introduction:**

Palliative care significantly improves the distressing symptoms of patients, especially those with cancer, heart disease, renal disease, and liver disease. The need for palliative care is increasing worldwide due to the growing burden of chronic disease. Nurses with an unfavorable attitude towards palliative care cannot skillfully assess the patient’s needs, do not communicate effectively, and do not address the patient’s problems adequately. Therefore, this study was aimed to assess the nurse’s level of attitude towards palliative care in Ethiopia.

**Methods:**

Several databases were searched to find available articles. Microsoft Excel was used to extract and sort the data before it was exported to STATA/MP 17.0 for analysis. A weighted inverse variance random-effects model with a 95% confidence interval was employed to pool the data. Egger’s test and Cochrane I^2^ statistics were used to assess heterogeneity and publication bias, respectively. Subgroup analysis was carried out to identify the source of heterogeneity. A log-odds ratio was employed to show the relationship between nurses’ level of attitude towards palliative care and its related factors. *P*-value less than 0.05 was considered statistically significant.

**Result:**

In Ethiopia, the pooled prevalence of favorable attitudes of nurses towards palliative care was 66.13% (95% CI: 54.00–78.27). The highest percentage of favorable attitudes towards palliative care among nurses was found in research studies done in Addis Ababa (80.31%; 95% CI: 72.00–88.63). Training on palliative care was significantly associated with the level of a nurse’s attitude towards palliative care. Therefore, nurses who received palliative care training had a 2.5 times higher chance of having a favorable attitude towards palliative care than nurses who did not receive training on palliative care (AOR = 2.55; 95% CI: 2.28–2.82).

**Conclusion:**

One-third of nurses had unfavorable attitude towards palliative care. Nurses who took palliative care training had a more favorable attitude than nurses who did not take palliative care training. Routine palliative care training is needed for nurses to improve their level of attitude towards palliative care.

**Supplementary Information:**

The online version contains supplementary material available at 10.1186/s12904-024-01402-w.

## Introduction

The primary responsibilities of nurses are to provide holistic and humanistic care that takes into account the patient’s surroundings, body, and soul [1]. Palliative care is one kind of comprehensive and humanistic nursing treatment [2]. Based on a critical analysis of the available definitions, palliative care is the care of a person with a life-threatening or substantially life-limiting disease, requiring treatment of physical or mental symptoms resulting from the disease or its treatment, providing support in the area of social and spiritual needs, culture, and sexuality, aimed at alleviating suffering and optimizing the quality of life of the person and their relatives, carried out regardless of disease activity and the treatment modifying its course in its early stages, terminal phase, during the dying, and after the patient’s death concerning the relatives [3]. It is also a pain management strategy that improves the quality of life for people who are suffering from a serious disease. It covers any type of nursing care intended to decrease the severity of symptoms [4]. Palliative care significantly improves the distressing symptoms of patients, especially those with cancer, heart disease, renal disease, and liver disease [5].

Globally, palliative care is becoming more necessary due to the rising burden of chronic illnesses [6]. There is a rise in the incidence, prevalence, and death rate of chronic illness, along with comorbidities and long-term disability, worldwide [7]. The need for effective palliative care services may become more significant in low-income countries, including Ethiopia [8]. There are different barriers to accessing palliative care in low-income countries. These are lack of resources, ignorance of palliative care, reluctance of nurses to give palliative care, and an insufficient number of nurses who are responsible for providing palliative care [9].

Nurses are holistic treatment providers at different levels of care (primary care, secondary care, tertiary care, and quaternary care) and act as a link between professionals, patients, and their families, all of which improve the standard of care for each patient [10]. They are crucial in delivering high-quality palliative care. The quality of palliative care is affected by nurses’ attitudes towards palliative care [11]. Nurses with a favorable attitude can decrease the suffering of patients and reduce their costs for hospitalization [12]. However, those nurses who have an unfavorable attitude are unable to communicate effectively, assess patients’ needs, and deal with their issues [13].

Previous studies showed that the percentages of nurses with favorable attitudes towards palliative care were 69.1% [14], 56.6% [15], 44.25% [16], and 53.41% [17]. The level of attitude among nurses may be influenced by different factors. These include experience, level of education, training on palliative care, knowledge about palliative care, and in-service training [18–20]. In Ethiopia, the issue of palliative care and the nurse’s level of attitude towards palliative care were not well discussed previously. Therefore, it is necessary to evaluate the attitude of nurses towards palliative care among Ethiopian nurses. Thus, this study is designed to evaluate nurses’ level of attitude towards palliative care in Ethiopia.

## Methods

### Study protocol

This systematic review and meta-analysis was conducted to evaluate the attitude of nurses towards palliative care and its associated factors in Ethiopia using the Preferred Reporting Items for Systematic Review and Meta-analysis (PRISMA) protocol for reporting findings (Table S1) [21].

### Daabases and searching strategies

In this systematic review and meta-analysis, several databases were searched. These include Google Scholar, Web of Science, African Journals Online (AJOL), HINARI, PubMed/MEDLINE, and EMBASE. In addition, unpublished articles from the repositories of Ethiopian universities were searched. The search terms were “attitude,” “feeling,” “perception,” “palliative care,” “end-of-life care,” “EOL,” “caring terminally ill,” “factors,” “associated factors,” “determinant factors,” “nurses,” “hospital-based nurses,” and “Ethiopia.” “AND” and “OR” Boolean operators’ strings were used (Table 1).

**Table 1:** Search of databases about Attitude of Nurses towards palliative care and its associated factors in Ethiopia

Databases	Searching terms	Number of studies
MEDLINE/ PubMed	“Attitude” OR “feeling” OR “perception” AND “palliative care” OR “PC” OR “EOL” OR “end of life care” OR “caring terminally ill” AND “factors” OR “associated factors” OR “determinant factors” AND “nurses” OR “hospital-based nurses” AND “Ethiopia”	236
Google Scholar	“Attitude” OR “feeling” OR “perception” AND “palliative care” OR “PC” OR “EOL” OR “end of life care” OR “caring terminally ill” AND “factors” OR “associated factors” OR “determinant factors” AND “nurses” OR “hospital-based nurses” AND “Ethiopia”	10,700
Other sources	“Attitude” OR “feeling” OR “perception” AND “palliative care” OR “PC” OR “EOL” OR “end of life care” OR “caring terminally ill” AND “factors” OR “associated factors” OR “determinant factors” AND “nurses” OR “hospital-based nurses” AND “Ethiopia”	4
Total retrieved articles		10,940
Included studies		11

### Screening and eligibility of the studies

The retrieved articles were exported to EndNote Reference software version 8 (Thomson Reuters, Stamford, CT, USA) citation manager to sort and avoid possible duplications. Three investigators (AG, MA, and AW) independently evaluated each study by title and abstract using predetermined inclusion criteria. The first name of the authors, publication year, region where the study was conducted, sample size, study period, the attitude of nurses, and factors affecting nurses’ attitudes towards palliative care were extracted. Any discrepancies between the authors during the process of extraction, evaluation, and reviewing of the articles were resolved. All studies reporting the level of attitude of nurses towards palliative care and its associated factors in Ethiopia, which were published until December 2023, were included. Articles that did not report outcome variables, qualitative studies, interventional studies, trials, case reports, news, and studies without full text were excluded from the analysis. Each author independently evaluates the eligibility of the articles.

### Outcome measurement of the study

The outcomes of this study are the attitude of nurses towards palliative care and its associated factors. The attitude of nurses was measured by the mean score of the Frommelt Attitudes Towards Care of the Dying (FATCOD) scale. Then, the outcomes were categorized as a favorable attitude and unfavorable attitude. Those nurses who were scored mean and above of the FATCOD scale were considered as having a favorable attitude, whereas nurses who were scored below the mean of the FATCOD scale were considered to have unfavorable attitude [22–24].

### Quality assessment

Three authors, AG, MA, and AW, independently evaluated the quality of each study using the Newcastle Ottawa Scale (NOS) for cross-sectional studies [25]. The methodological quality, comparability, outcomes, and statistical analysis of the studies were the assessment tools used to declare the quality of studies. Studies scored on a scale of > 7 out of 10 were considered as achieving high quality. All authors independently assessed the articles for consideration and inclusion in the final analysis.

### Data processing and analysis

The data was extracted and cleaned using a Microsoft Excel spreadsheet. It was exported to STATA version 17 for analysis. The inverse variance random-effects model at 95% CI was used to weigh the pooled prevalence of nurses’ attitudes towards palliative care and its associated factors in Ethiopia [26]. The Cochrane Q-test and I^2^ with the correspondence *p*-value were used to evaluate the studies’ heterogeneity [27]. Subgroup analysis was carried out by the study region to investigate the possible cause of heterogeneity. Sensitivity analysis was carried out to check the presence of influential studies. Additionally, Egger’s test was carried out to look for publication bias and displayed with a funnel plot [28]. A log odds ratio was used to determine the association between the associated factors and the nurse’s level of attitude towards palliative care. A statistical test with a *P*-value of < 0.05 was considered statistically significant.

## Result

**Fig. 1:**
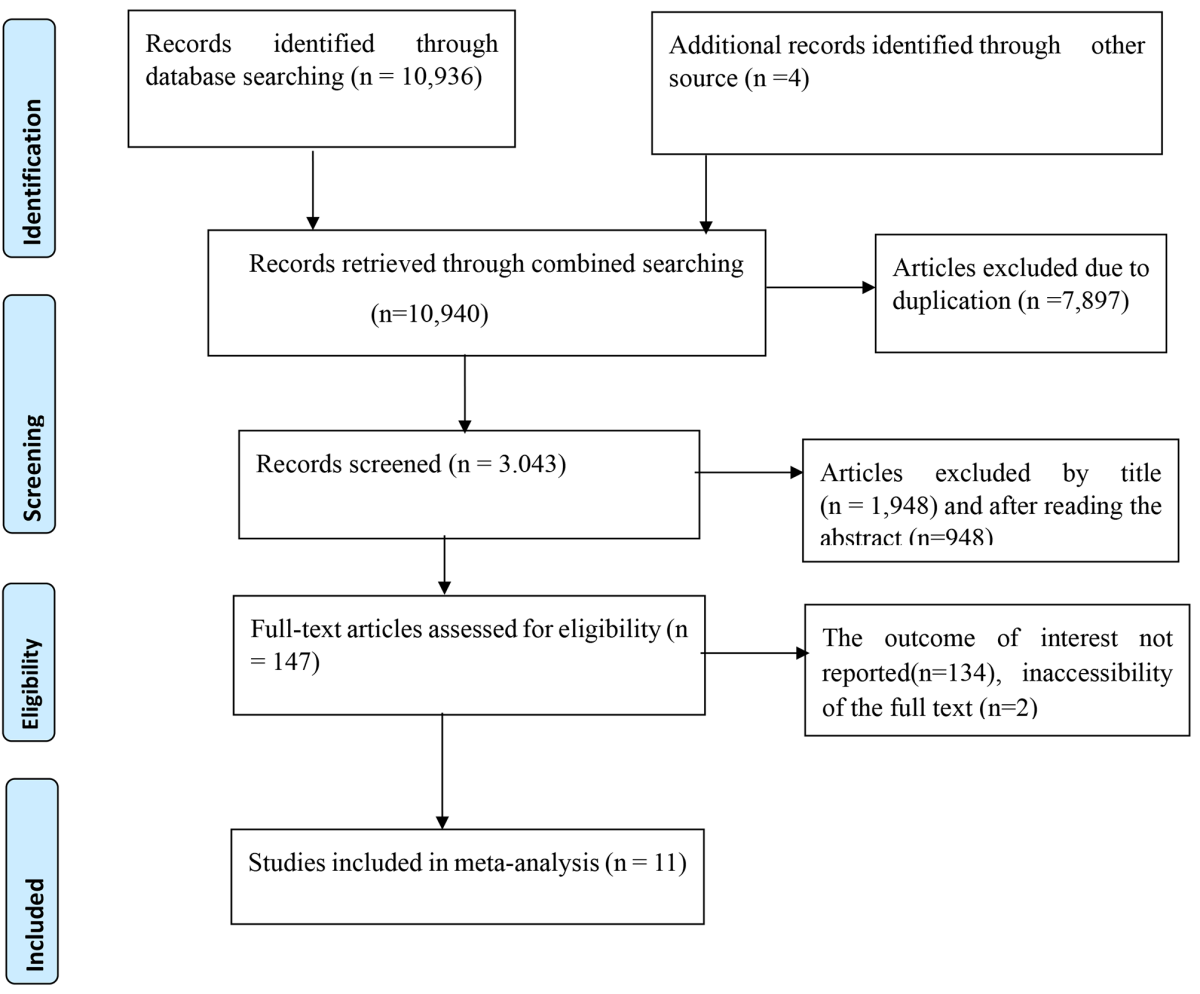
Flow chart of selection articles done on attitude of nurses towards palliative care and its associated factors in Ethiopia

In this study, 10,940 articles were retrieved from different databases. Of these articles, 7,897 were excluded due to duplication. In addition, 2,896 articles were removed after reviewing the titles and abstracts of the studies. Furthermore, 134 articles were excluded that did not fulfil the inclusion criteria. Two articles were also excluded due to an inability to get the full text. Finally, eleven articles were included in the final analysis (Fig. 1).

### Characteristics of the studies and study participants

This systematic review and meta-analysis covered eleven studies published up until December 2023, involving 3,468 study participants. From the included studies, four were from the Amhara region [16, 17, 29, 30], two from Addis Ababa city administration [31, 32], two from the Tigray region [33, 34] and three from the Oromia region [24, 35, 36]. All studies were cross-sectional in design and the sample size of the included studies was found in the rage of 197–392 (Table 2).

**Table 2:** Characteristics of studies and study participants on attitude of nurses towards palliative care and its associated factors in Ethiopia

Author/Publication year	Region	Sample size	Favorable attitude (%)	Level of education (%)		Work experience in year (%)			Experience in caring chronically ill patients (%)					Training on palliative care (%)		Good knowledge about palliative care (%)
				Diploma	Degree	< 5	5–10	> 10	Daily	Once /week	Once/month	Few/year	Never	Yes	No	
Abate et al., 2019	Amhara	331	70.7	13.6	86.4	69.5	23.3	7.3	46.2	23.6	14.5	1.2	14.5	18.7	81.3	38.97
Getie A et al., 2020	Amhara	226	44.2	41.2	58.8	64.2	28.8	7.1	35.4	11.9	17.3	15.9	19.5	26.1	73.9	59.73
Anteneh S et al., 2016	Amhara	352	53.4	42.0	58.0											53.13
Tesfaye et al., 2018	Oromia	237	79.3	44.7	55.3											58.23
Zeru et al., 2020	Tigray	355	56.3	47.6	52.4	38.3	29.6	32.1	46.5	22.3	4.8	11.3	15.2	75.2	24.8	62.82
Gedamu S et al., 2019	Addis Ababa	392	84.4	14.5	85.5	64.0	24.7	11.2	69.1	14.3	4.6	9.4	2.6	28.6	71.4	26.53
Kassa H et al., 2014	Addis Ababa	341	76.0	49.9	50.1	53.4	20.5	26.1	54.5	20.5	7.9	9.7	7.3	21.7	78.3	30.50
Meaza D et al., 2015	Oromia	197	88.3			53.3	20.8	25.9						29.4	70.6	55.84
Aytenew et al., 2022	Amhara	387	29.5	37.5	62.5	56.8	33.6	9.6							100	21.45
Etafa W et al., 2020	Oromia	372	50.8	18.5	81.5	52.7	34.1	13.2								51.88
Zeru et al., 2020	Tigray	278	93.9	43.2	56.8	43.2	27.0	29.9	41.4	22.3	7.9	9.0	0.0			25.18

### Attitude of nurses towards palliative care

**Fig. 2:**
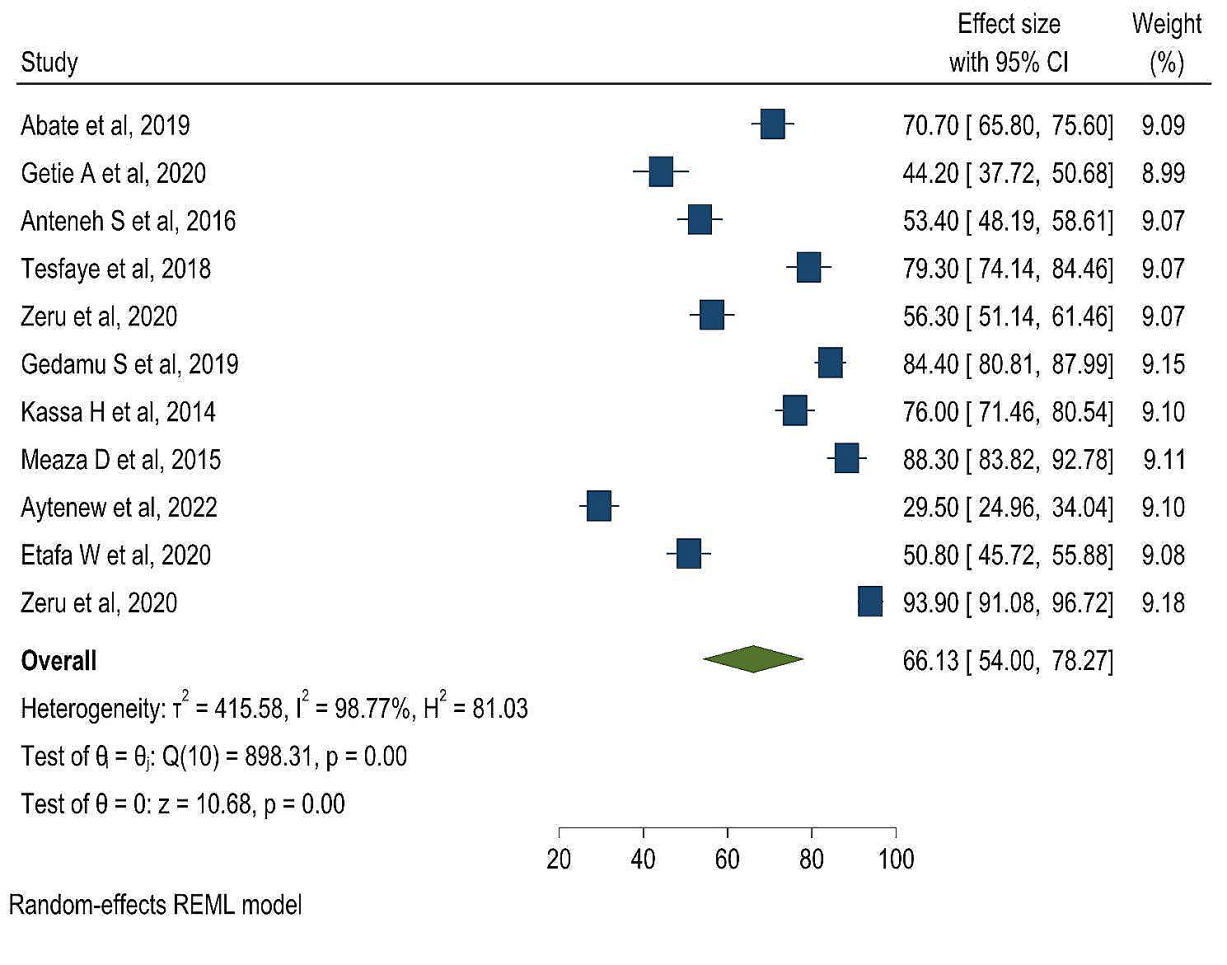
Frost plot on the pooled prevalence of favorable attitude of nurses towards palliative care in Ethiopia

This systematic review and meta-analysis showed that, the pooled prevalence of favorable attitude of nurses towards palliative care in Ethiopia was 66.13% (95% CI: 54.00-78.27) (Fig. 2).

### Heterogeneity and publication bias

In this systematic review and meta-analysis, there is a high heterogeneity within the studies (I^2^ 98.77%, *p* < 0.001). The Egger’s test revealed a statistically significant result (*p* = 0.015), indicating the possibility of publication bias.

### Sub-group analysis

**Fig. 3:**
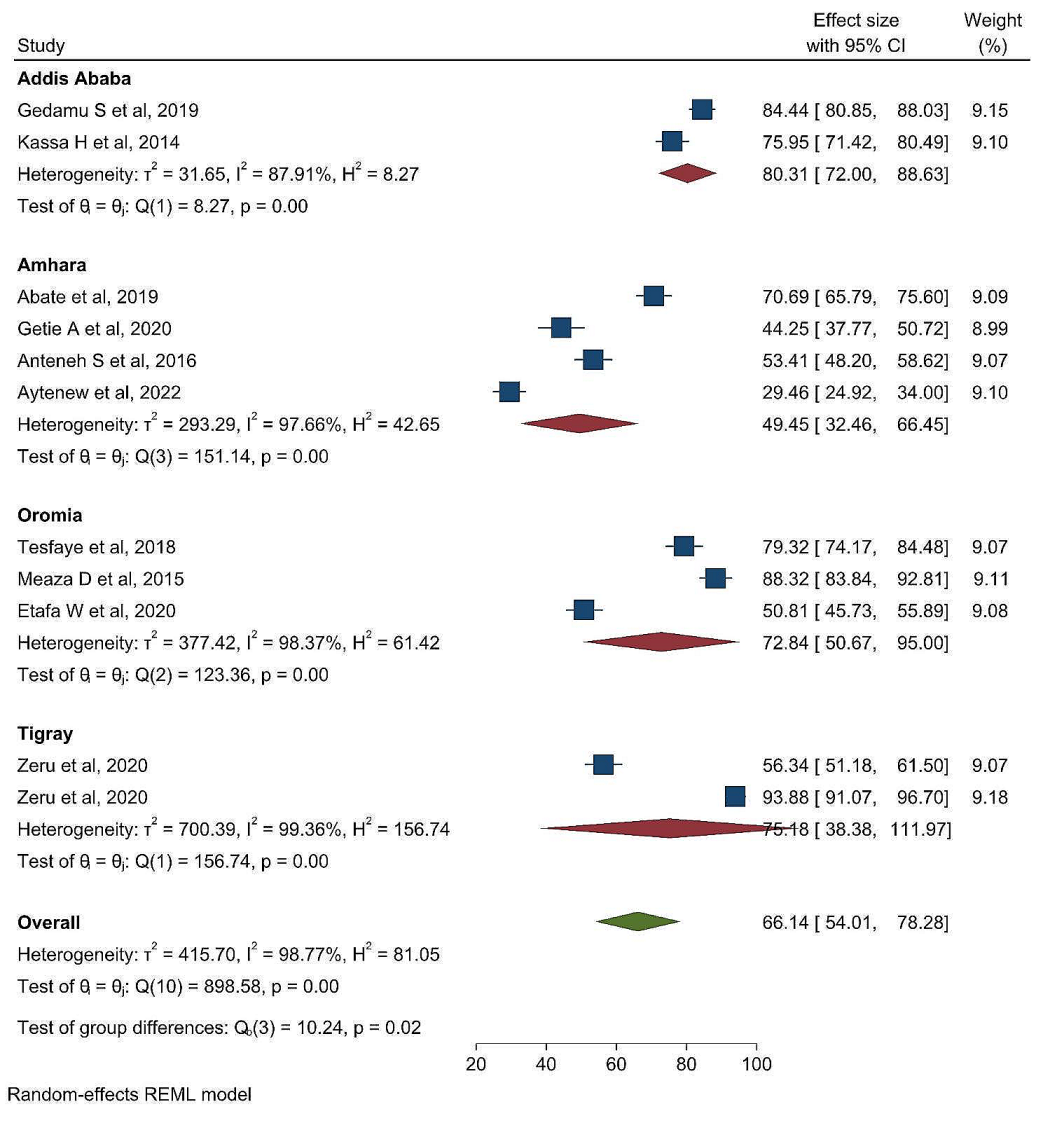
Sub-group analysis by region on the pooled prevalence of favorable attitude of nurses towards palliative care in Ethiopia

Sub-group analysis was performed by the region where the studies were done to detect the source of heterogeneity. Research studies conducted in Addis Ababa revealed the highest percentage of favorable attitude of nurses towards palliative care in Ethiopia: 80.31% (95% CI:72.00-88.63), where as the lowest was reported in Amhara region 49.45% (95% CI: 32.46–66.45) (Fig. 3).

### Sensitivity analysis

A leave-one-point sensitivity analysis conducted using the random-effects model revealed that all of the points were estimates within the overall 95% confidence interval (54.00-78.27) indicating the absence of any influential study.

### Work experience, level of education, experience on caring chronicaly ill patients and training on palliative care

In this study, the majority of nurses 55.06% (95% CI: 48.46–61.67) had less than five years of work experience. Nurses who had a BSc. degree were 64.87% (95% CI: 56.06, 73.67). Similarly, nurses who had a daily experience of caring chronically ill patients were 48.98% (95% CI: 39.50–58.40). In addition, 66.71% (95% CI: 48.73, 84.68) of nurses did not receive training on palliative care. Furthermore, 43.92% (95% CI: 34.62, 53.22) of nurses had good knowledge on palliative care (Table 3).

**Table 3:** Work experience, level of education, experience on caring chronicaly ill patients, training on palliative care, and level of knowledge about palliative care among nurses working in Ethiopia

Variables	Classifications	Studies	Prevalence (95%CI)	I^2^ (%)	*P*-value
Work experience	Less than five years	9	55.06 (48.46,61.67)	92.66	< 0.001
	Five to ten years	9	26.92 (23.59,30.24)	76.33	< 0.001
	Greater than ten years	9	17.84 (11.16,24.52)	96.53	< 0.001
Level of education	Diploma	10	35.13 (26.33,43.94)	97.04	< 0.001
	BSc (degree)	10	64.87 (56.06,73.67)	97.07	< 0.001
Experience in caring chronically ill patients	Daily	6	48.98 (39.50,58.40)	94.64	< 0.001
	Once/week	6	19.17 (15.31,23.02)	96.43	< 0.001
	Once/month	6	09.19 (05.18,13.21)	91.22	< 0.001
	Few/year	6	09.14 (05.25,13.02)	91.67	< 0.001
	Never	6	11.56 (05.59,17.53)	91.67	< 0.001
Training on palliative care	Yes	6	33.29 (15.32,51.27)	98.80	< 0.001
	No	6	66.71 (48.73,84.68)	98.80	< 0.001
Level of knowledge on palliative care	Good knowledge	11	43.92 (34.62,53.22	97.18	< 0.001

### Factors associated with level of nurses attitude towards palliative care

**Fig. 4:**
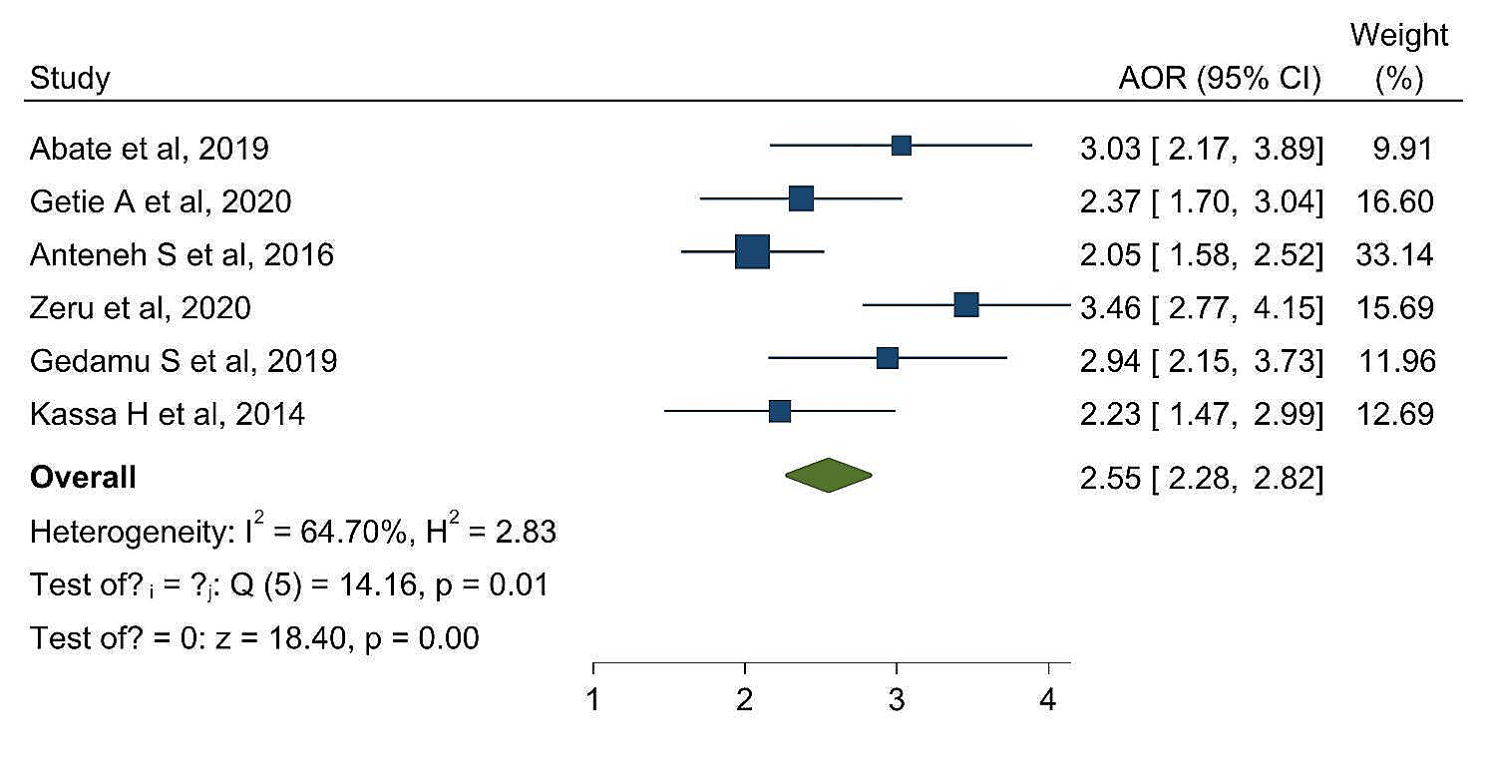
The overall pooled odds ratio of the association beteween training on palliative care and nurses attitude towards palliative care in Ethiopia

The results of this systematic review and meta-analysis indicate a significant association between nurses’ level of attitude towards palliative care and palliative care training. Then, nurses who had received palliative care training had a 2.5 times higher chance of having a favorable attitude towards palliative care than nurses who did not receive palliative care training (AOR = 2.55; 95% CI: 2.28–2.82) (Fig. 4).

## Discussion

This systematic review and meta-analysis evaluate the level of nurses’ attitudes towards palliative care in Ethiopia. The findings of this study showed that 66.13% (95% CI: 54.00–78.27) of nurses had a favorable attitude towards palliative care. Similar findings were reported in different countries: 56.6% in Sudan [15], 69.1% in Mongolia [14], 62.4% in Palestine [37], and 58.9% in Democratic Republic of Congo [38]. In this study, the level of favorable attitude of nurses towards palliative care was lower than studies conducted in Iran (81.8%) [39] and India (92.8%) [40]. This difference might be because of cultural differences related to giving care to dying patients and the difference in case flow. Previous studies were conducted in high-income countries, where nurses have more exposure to patients who need palliative care. This builds their level of attitude towards palliative care. It might also be due to the absence of curriculum education content about palliative care in Ethiopia. However, the level of favorable attitude of nurses in Ethiopia was higher than in a study done in Egypt (337.6% of nurses had a positive attitude towards palliative care) [41]). This discrepancy might be due to the differences in in-service training, knowledge about palliative care, formal palliative care education, and job satisfaction of nurses [16, 29].

In this systematic review and meta-analysis, there is a high heterogeneity within the studies (I^2 =^ 98.77%, *p* < 0.001). To detect the possible source of heterogeneity, subgroup analysis by region was conducted. Research studies conducted in Addis Ababa revealed the highest percentage of favorable attitudes of nurses towards palliative care: 80.31% (95% I: 72.00–88.63). This could be because of the difference in study settings; in Addis Ababa, nurses worked in specialized and referral hospitals, where nurses routinely encountered and managed patients in need of palliative care. In addition, nurses that were recruited in hospitals found in Addis Ababa had the chance to get training on palliative care. Therefore, frequent exposure to chronically and terminally ill patients and getting training in palliative care increase the development of a favorable attitude towards palliative care. This study found a significant association between nurses’ level of attitude towards palliative care and palliative care training. Nurses who received palliative care training had 2.55 times higher odds of having a favorable attitude towards palliative care than nurses who did not receive palliative care training. One possible explanation is that well-trained nurses tend to have positive attitudes due to their strong expertise [33, 42, 43].

### Strength and limitation of the study

This study highlights the nationwide picture of level of nurse’s attitude towards palliative care in Ethiopia. It covers a wide area and investigates different articles, making the review more accurate. Subgroup and sensitivity analyses were carried out to investigate the heterogeneity of the included studies. However, studies whose study design cross-sectional were limit investigation of the cause–effect relationship.

## Conclusion

In Ethiopia, two-thirds of nurses had a favorable attitude towards palliative care. There was regional variation regarding the level of nurses’ attitude towards palliative care; the highest level of favorable attitude was reported among nurses who worked in Addis Ababa, whereas the lowest was reported in the Amhara region. Palliative care training was significantly associated with nurses’ level of attitude towards palliative care. Accordingly, the level of favorable attitude was higher among nurses who took palliative care training than among those who did not take palliative care training. Thus, palliative care training and improving nurses’ careers through continuous professional development should be given regularly to nurses to improve their level of attitude towards palliative care.

### Electronic supplementary material

Below is the link to the electronic supplementary material.

Supplementary Material 1

## Data Availability

All related data have been presented within the manuscript. The dataset supporting the conclusions of this article is available from the authors on request.

## Abbreviations

AJO: African Journals Online
AOR: Adjusted Odds Ratio
BSc: Bachelor of Science
CI: Confidence Interval
FATCOD: Frommelt Attitudes Toward Care of the Dying
NOS: Newcastle Ottawa Scale
PRISMA: Preferred Reporting Items for Systematic Review and Meta-analysis
